# Optimized radiofrequency shimming using low-heating B_1_^+^-mapping in the presence of deep brain stimulation implants: Proof of concept

**DOI:** 10.1371/journal.pone.0316002

**Published:** 2024-12-18

**Authors:** Maryam Arianpouya, Benson Yang, Fred Tam, Clare E. McElcheran, Simon J. Graham

**Affiliations:** 1 Physical Sciences Platform, Sunnybrook Research Institute, Toronto, ON, Canada; 2 Department of Medical Biophysics, University of Toronto, Toronto, ON, Canada; 3 TECHNA Institute for the Advancement of Technology for Health, Toronto, ON, Canada; King’s College London, UNITED KINGDOM OF GREAT BRITAIN AND NORTHERN IRELAND

## Abstract

MRI of patients with Deep Brain Stimulation (DBS) implants is constrained due to radiofrequency (RF) heating of the implant lead. However, “RF-shimming” parallel transmission (PTX) has the potential to reduce DBS heating during MRI. As part of using PTX in such a “safe mode”, maps of the RF transmission field (B_1_^+^) are typically acquired for calibration purposes, with each transmit coil excited individually. These maps often have large zones of low signal intensity distant from the specific coil that is being excited, raising concerns that low signal-to-noise ratio (SNR) in these zones might negatively impact the ability of the optimized RF shim settings to suppress heating in safe mode. One way to improve SNR would be to increase RF transmission power during B_1_^+^ mapping, but this also raises heating concerns especially for coil elements proximal to the implant. Acting with an abundance of caution, it would be useful to investigate methods that permit B_1_^+^ mapping with low localized heating while producing high SNR measurements that lead to safe PTX RF shim settings. The present work addresses this issue in proof of concept using electromagnetic simulations and experimental PTX MRI. A two-step optimization algorithm is proposed and examined for a cylindrical phantom with an implanted wire to enable 1) robust B_1_^+^ mapping with low localized heating; and 2) robust RF shimming PTX with low localized heating and good B_1_^+^ homogeneity over a large imaging volume. Simulation and experimental outcomes were compared with those obtained using an existing simulation-driven workflow for obtaining safe mode RF shim settings, and for quadrature RF transmission using a circularly polarized (CP) birdcage head coil. Experimental results showed that although both existing and proposed safe-mode workflows effectively suppressed localized heating at the wire tip in comparison to the CP coil results, the proposed workflow produced much smaller temperature elevations and much improved signal uniformity. These promising results support continued investigation and refinement of the proposed workflow, involving more realistic scenarios toward ultimate implementations in DBS patients.

## Introduction

Magnetic resonance imaging (MRI) of patients with deep brain stimulation (DBS) implants is significantly constrained due to localized radiofrequency (RF) heating, primarily surrounding the electrode tip of the implant. The heating, quantified by the local specific absorption rate (SAR), is caused by the electric field (E-field) component of the RF transmission during MRI. The scattered E-field is concentrated in tissue surrounding the DBS lead tip [1, 2] and can also occur along the elongated lead extension where sharp bending exists [3, 4], with much less concern at the neurostimulator (IPG) housing [4]. Minimization of the incident E-field was initially introduced to mitigate RF heating near the implant, by utilizing the linear excitation mode of a birdcage coil to direct a zero (or “null”) E-field plane region towards the implant lead tip [5]. The same research group then demonstrated the utility of this concept using a dual-drive birdcage coil and a copper wire placed under porcine skin and muscle tissue [6].

The concept of E-field modification has subsequently been exploited and enhanced through the implementation of parallel radiofrequency transmission (PTX) techniques employing either a) a fixed input RF waveform and channel-specific phase or magnitude (i.e., “RF shimming”); or b) employing completely different input waveforms for each channel (i.e., “Full PTX”). An RF-shim method was demonstrated for nulling RF-induced currents in elongated metal structures using two-channel PTX [7], which was subsequently investigated in guidewires using a four-channel PTX arrangement [8]. Other studies [9–11] provided additional demonstration of the utility of RF-shim PTX involving two-, four- and eight- channel PTX coils, focusing on E-field minimization while attempting to maintain a spatially uniform magnetic transmission field (B_1_^+^); and by using the scattered B_1_^+^-field, as obtained by MRI, as a surrogate of induced current on the electrode [12]. Full PTX approaches, particularly involving “spokes” RF pulse designs, have also been investigated to maximize the spatial uniformity of RF transmission with hard constraints on global and local SAR. One study [13] demonstrated a spoke pulse design technique with electromagnetic (EM) simulations that produced uniform magnitude least-squares flip angle excitations with low E-fields near the implant. Another [14] utilized a similar method on eight- and sixteen-channel PTX coils with a single spoke in excitation spatial frequency (k) space, computing the power absorbed for MRI at 3 T in soft tissues surrounding the DBS implant tip; it was found that an eight-channel PTX coil reduced the local SAR value by a factor of 18 compared to a birdcage coil setup, at no cost to RF transmission spatial uniformity or global SAR.

Notably, other options are also being explored towards safe MRI of DBS patients. For example, materials have been introduced that are MRI-compatible, novel and safer in the DBS device context, such as poly (3,4-ethylenedioxythiophene) [15], graphene fibre [16] and semiconductor electrodes [17, 18]. Alternative lead designs, such as the “RF shield trap” [19] and high impedance circuits [20, 21] have also been conceived that are resistant to heating. To date, however, none of these technologies has translated to full clinically-approved and regulatory-approved DBS devices. It is reasonable, therefore, to continue exploring and expanding the capabilities of the “safe-mode” PTX MRI approach, towards applications involving existing and legacy MRI-conditional DBS devices.

The existing workflow for safe-mode PTX MRI commonly adopts the following pattern. For calibration purposes, the standard B_1_^+^-field profiles of the individual RF coil elements, known as transmission sensitivity maps, are determined by successively passing a current through one element and zero current through the others. (Note that in cases where safe mode is estimated solely using EM simulations and then applied in actual MRI measurements, both B_1_^+^-field and E-field maps can be obtained straightforwardly using the analogous procedure.) The sensitivity maps are then applied either to a) an optimization algorithm to find the best RF-shim weighting factors; or b) an RF pulse design optimization algorithm to find the best Full-PTX waveforms to minimize E-field or B_1_^+^ artifact intensity in areas with safety concerns (e.g. the lead tip). Some studies have included B_1_^+^ variation into the RF shim cost function to maintain adequate spatial homogeneity of the B_1_^+^-field over the imaging field of view (FOV) while minimizing the local E-field [9, 11, 22]. Last, the PTX solution is then applied on a real MRI system to achieve safe imaging conditions with reasonable image quality.

Although these procedures have been shown to be advantageous, there are also some limitations to address, and the present work is motivated by this consideration. One important aspect is that the B_1_^+^ sensitivity maps could be improved to make more accurate measurements throughout the FOV. To date, much of the research on B_1_^+^ mapping has focused on making these calibration measurements as quickly as possible for clinical purposes, because the number of maps required typically scales with the number of parallel transmission channels. This need for rapid calibration, and concerns that high-power “pre-scan” procedures could potentially cause undesirable heating effects in DBS patients [3, 23]—especially for PTX coil elements closest to DBS electrodes—have led to common use of low-power B_1_^+^ mapping approaches [12, 14]. Under these conditions, however, the standard approach of mapping the B_1_^+^ transmission profile of each coil element sequentially produces large zones of low signal intensity distant from the element that is being excited. The low signal-to-noise ratio (SNR) in these regions potentially can have a negative impact on the accuracy of the subsequent RF shim results [24, 25]. Due to the concerns over localized heating it is reasonable to use an abundance of caution and, borrowing a term from the field of radiation treatment, investigate how to keep localized heating near DBS implants “as low as reasonably achievable (ALARA)” while performing improved B_1_^+^ mapping throughout the FOV towards optimal safe-mode PTX MRI.

A B_1_^+^ mapping procedure has previously been developed in the absence of DBS implants that involves transmitting with different combinations of PTX channels, rather than single channels, thus reducing the zones of low signal intensity and improving SNR over large regions in the imaged object [24]. The aim of the present study is thus to extend this procedure to the DBS context, by applying it to an existing safe-mode PTX workflow (previously developed by some of the co-authors) which is heavily reliant on EM simulations [23]. The proposed new workflow consists of a two-step optimization algorithm. First, a low-SAR optimization method is developed for mapping transmission sensitivity in an MRI pre-scan procedure, using appropriate combinations of transmit channels. The low-SAR sensitivity maps are then used in a second optimization step that involves jointly minimizing a) excitation artifact at the DBS electrode tip (as a surrogate of localized SAR [12, 26, 27]) and b) B_1_^+^ inhomogeneity over a prescribed FOV. The new workflow is hypothesized to yield improved, more robust experimental results (less localized heating and improved image signal uniformity) in comparison with the existing method, and to suppress localized heating markedly in comparison with standard birdcage coil technique in which no optimization approach is conducted.

## Theory

In the current research context, certain prior information is important to generate PTX safe mode. Current practice for DBS implantation in many clinics includes intra-operative computed tomography (CT). Patient-specific “models” can thus be determined using these images and a tissue-mimicking medium (e.g. a typical gelatin-based brain model with heterogeneous grey and white matter segmentations [10], or more sophisticated realistic brain structures based on preoperative patient MRI [28]) to minimize discrepancy between simulation and actual MRI data. In addition, the EM model of the PTX coil that is used to generate safe mode can be obtained straightforwardly if, as in the present case, the investigators have developed their own custom coil design. (The information may also be possible to obtain if the coil is custom-built for the investigators by another research group, or if it is built by MRI vendors or third-party MRI technology companies. The latter scenario may be difficult to arrange in practice if the coil information is proprietary rather than open-source, however, likely requiring the various parties to enter into a legal agreement. Alternatives in this scenario include disassembling the coil for inspection, although this may invalidate the existing warranty, or analyzing internal and external features without disassembly using CT [29]). Once the EM model of the PTX coil is obtained, it can be reused for imaging all subsequent DBS patients as needed. The coil model can then be input to EM simulations, together with each patient model and simulated DBS lead trajectory (again, obtaining EM model information about the device as generally outlined above), to estimate transmission sensitivity maps and subsequently estimate settings for PTX safe-mode MRI.

Examples of this workflow, including sophisticated simulation of DBS device leads, electrodes and wiring, can be seen in work presented by various research groups [11, 30]. In what follows, a new two-step optimization algorithm is proposed (and tested in proof of concept) that builds on the existing workflow by incorporating a calibration procedure with improved regional SNR in B_1_^+^ mapping while keeping localized heating ALARA, towards eventual PTX safe-mode MRI of DBS patients.

### (1) Low SAR B_1_^+^ mapping

For PTX using RF shimming, the input signal (*S*_*n*_) to each channel (*n*) is achieved by a common waveform *S*(*t*) (representing the amplitude-modulated envelope of the RF-transmitted waveform; with a “sinc” envelope commonly used for image slice selection) multiplied by channel-dependent relative amplitude (*A*_*n*_) and phase (*ϕ*_*n*_) factors:

$$S_{n}(t)=A_{n}e^{i\phi_{n}}S(t).$$ (1)

The total B_1_^+^-field and E-field at each point in the FOV can be calculated as the sum of the amplitude- and phase-adjusted, channel-dependent B_1_^+^- and E- field transmission sensitivity maps, respectively. By selecting the optimized weighting factors *A*_*n*_ and *ϕ*_*n*_ for each channel, the desired B_1_^+^- and E-fields can be achieved spatially as follows in EM simulations. The standard maps (i.e. driving only one channel at a time) are obtained according to a simple matrix formalism [23, 24] involving *S*(*t*) multiplied by a basis set ***C*** of orthogonal unity vectors that represent PTX “channel coordinates” (e.g. for 4-channel PTX: S(t)×([1,0,0,0],[0,1,0,0],[0,0,1,0],[0,0,0,1])). This yields three insights from linear algebra: a) the sensitivity maps span all possible EM field distributions that can be generated by the coil; b) other orthogonal basis sets must exist with the same spanning property; and c) switching from one basis set to another is achievable using a simple matrix transformation. Therefore, the goal becomes to find a basis set of “low-SAR coordinates” which avoids substantial localized heating of DBS electrodes and yields sensitivity maps that contain less extensive regions with low signal intensity that are prone to noise contamination.

To achieve this, the simulated standard sensitivity maps are input to a constrained optimization algorithm to create a matrix ***L*** of low-SAR basis vectors of complex weights ($L_{n,m}={A_{n}}^{\left(m\right)}e^{i{\phi_{n}}^{\left(m\right)}}$) for B_1_^+^ -mapping, minimizing the absolute square of the total E-field, |*E*_*tot*_|^2^ (and thus SAR) over a specified region of minimization (ROM) surrounding the DBS lead tip:

$$\underset{{A_{n}}^{\left(m\right)},{\phi_{n}}^{\left(m\right)}}{\min}\left(\mu_{ROM}({\left|E_{tot}\right|}^{2})\right),$$ (2A)

where $\left({A_{n}}^{\left(m\right)},{\phi_{n}}^{\left(m\right)}),({A_{n}}^{\left(m-1\right)},{\phi_{n}}^{\left(m-1\right)}),\ldots ,({A_{n}}^{\left(0\right)},{\phi_{n}}^{\left(0\right)})\;\{n,m=1\ldots N\right\}$ represent the *m*_*th*_ elements of the *n*_*th*_ vector of inputs to each coil element; *μ*_ROM_(∙) is the spatial average over the ROM, chosen as the 1-gram (SAR_1g_) region of tissue surrounding the implant tip; and very importantly, the basis vectors are determined subject to specific orthogonality constraints:

$$\frac{(m-1)\pi }{2N}+Q\leq P({\phi_{n}}^{\left(m\right)},N)<\frac{m\pi }{2N}+Q$$ (2B)

$$\frac{0.2(N+m-1)}{N}+M\leq P({A_{n}}^{\left(m\right)},N)<\frac{0.2(N+m)}{N}+M$$ (2C)

where $Q=\frac{2\pi (n-1)}{N}$ and $M=\frac{0.8(n-1)}{N}$ are the quadrature terms for creating orthogonal amplitudes and phase vectors. The term *P* returns all possible orthogonal permutations of a basis set to fulfill the desired spanning of all EM field distributions.

For practical considerations, additional constraints are also included for Eqs (2A)–(2C), consisting of lower and upper bounds on the *A*_*n*_ values. As very small *A*_*n*_ may result in B_1_^+^ maps with poor SNR characteristics in actual MRI experiments, the lower bound was set at 0.2. As very large *A*_*n*_ may produce an *L*^−1^ matrix with a low condition number, the upper bound was set at the maximum value of 1.0. The *L*^−1^ matrix is used for transforming safe-mode PTX settings in low-SAR coordinates to channel coordinates (see below for details) and, if chosen poorly, this transformation has the potential to introduce significant noise amplification in the ultimate safe-mode PTX settings applied at the MRI system console. The amount of noise amplification is typically quantified by the matrix condition number, which in the ideal case has a value of 1, indicating no noise amplification [31]. Conversely, an ill-conditioned matrix producing highly erroneous PTX settings has a condition number of infinity. Given this range, the chosen upper bound of 1.0 on *A*_*n*_ values is a reasonable constraint against noise amplification, suitable for the present proof-of-concept work. Lastly, the sets of amplitudes were permuted during optimization to achieve a good span of all solution estimates for *A*_*n*_ between 0.2 and 1. Permutation of the amplitudes helps to prevent the optimization procedure from becoming “trapped” at local minima.

For example, the ***L*** matrix of a 4-channel PTX system has the following configuration:

$$\begin{array}{cccc} n1 & n2 & n3 & n4 \end{array}$$

$$\begin{array}{c} m1\\ m2\\ m3\\ m4 \end{array}\left[\begin{array}{cccc} A_{1,1},\phi_{1,1} & A_{1,2},\phi_{1,2} & A_{1,3},\phi_{1,3} & A_{1,4},\phi_{1,4}\\ A_{2,1},\phi_{2,1} & A_{2,2},\phi_{2,2} & A_{2,3},\phi_{2,3} & A_{2,4},\phi_{2,4}\\ A_{3,1},\phi_{3,1} & A_{3,2},\phi_{3,2} & A_{3,3},\phi_{3,3} & A_{3,4},\phi_{3,4}\\ A_{4,1},\phi_{4,1} & A_{4,2},\phi_{4,2} & A_{4,3},\phi_{4,3} & A_{4,4},\phi_{4,4} \end{array}\right].$$

The ***L*** matrix basis vectors (***m1***, ***m2***, ***m3***, ***m4***) can then be applied at the MRI system console to acquire the associated low-SAR B_1_^+^-maps of transmission sensitivity. These maps are then utilized in step 2 of the proposed new workflow, reflecting the actual PTX hardware better than can be achieved using the simulation model alone.

### (2) Minimizing B_1_^+^-field inhomogeneity with low local SAR

Because the low-SAR B_1_^+^ -maps span the field space, a second, more complex optimization can next be performed to find the best sets of amplitude and phase settings (*A*′_*m*_ and *φ*′_*m*_) in the ***L*** basis which jointly minimize local SAR (through the $B_{1}^{artifact+}$ term, explained below) and B_1_^+^ non-uniformity:

$$\underset{{A\prime }_{m},{\varphi \prime }_{m}}{\min}(\frac{\sigma_{ROI}(\left|B_{1,tot}^{+}\right|)}{\mu_{ROI}(\left|B_{1,tot}^{+}\right|)}+\lambda ∙\mu_{ROM}\left(|B_{1}^{artifact+}|\right)),$$ (3)

where *σ*_ROI_ and *μ*_ROI_ are the spatial standard deviation and average over a chosen imaging region of interest (ROI), and λ is a real regularization factor used to control the relative importance of the $B_{1}^{artifact+}$ term, constrained to take on any value between 0 (no importance) and 1 (equal importance).

The $B_{1}^{artifact+}$ term is important when considering a practical implementation of such optimization procedures in actual patients. A direct calculation of SAR requires imaging of the E-field, but this remains very challenging using MRI methods [32, 33]. Another parameter in the SAR calculation—the electrical conductivity—is also difficult to estimate by imaging [34]. However, others have proposed a surrogate for SAR values that is useful in the present context [26, 27]. The induced current in the electrode, *I*_*ind*_, that is responsible for localized heating during MRI also produces similarly localized image artifacts. According to Ampere’s law, *I*_*ind*_ creates a scattered B-field in the metallic lead which couples with the transmitted RF B_1_^+^-field and produces artifact. The artifact can be determined by subtracting the “background” field $B_{1,coil}^{+}$ (when no wire is present, i.e., the induced current *I*_*ind*_ is zero) from the total B_1_^+^ field, $B_{1,tot}^{+}$, in the ROM:

$${\hat{B}}_{1}^{artifact+}={\hat{B}}_{1,tot}^{+}-{\hat{B}}_{1,coil}^{+}.$$ (4)

The ${\hat{B}}_{1,coil}^{+}$ can be estimated experimentally from the acquired B_1_^+^ maps by making one or more-point measurements proximal to the artifact–in the present case, for example, simply by interrogating the nearest-neighbour transversal slice above the implant tip. By substituting Eq (4) in Eq (3):

$$\underset{{A\prime }_{m},{\varphi \prime }_{m}}{\min}(\frac{\sigma_{ROI}(\left|B_{1,tot}^{+}\right|)}{\mu_{ROI}(\left|B_{1,tot}^{+}\right|)}+\lambda ∙\mu_{ROM}\left(|{\hat{B}}_{1,tot}^{+}-{\hat{B}}_{1,coil}^{+}|\right)).$$ (5)

After the two optimizations are performed (Eqs (2A)–(2C) and (5)), the vectors of obtained amplitude and phase shifts ($W_{m}={A\prime }_{m}e^{i{\varphi \prime }_{m}}$) for safe mode in the low-SAR basis are then transformed to provide actual RF shimming settings at the coil inputs ($U_{n}=A_{n}e^{i\varphi_{n}}$):

$$U=L^{-1}W,$$ (6)

where $U={\left[U_{1}\;U_{2}\cdots U_{N}\right]}^{T}$ is a matrix of the actual RF shimming settings, $W={\left[W_{1}\;W_{2}\cdots W_{N}\right]}^{T}$ is a matrix of the complex weighting factors obtained through optimization in the low-SAR basis, and ***L*** is as defined previously. By design, ***L*** is invertible allowing ***U*** to be easily obtained.

## Methods

**Fig 1:**
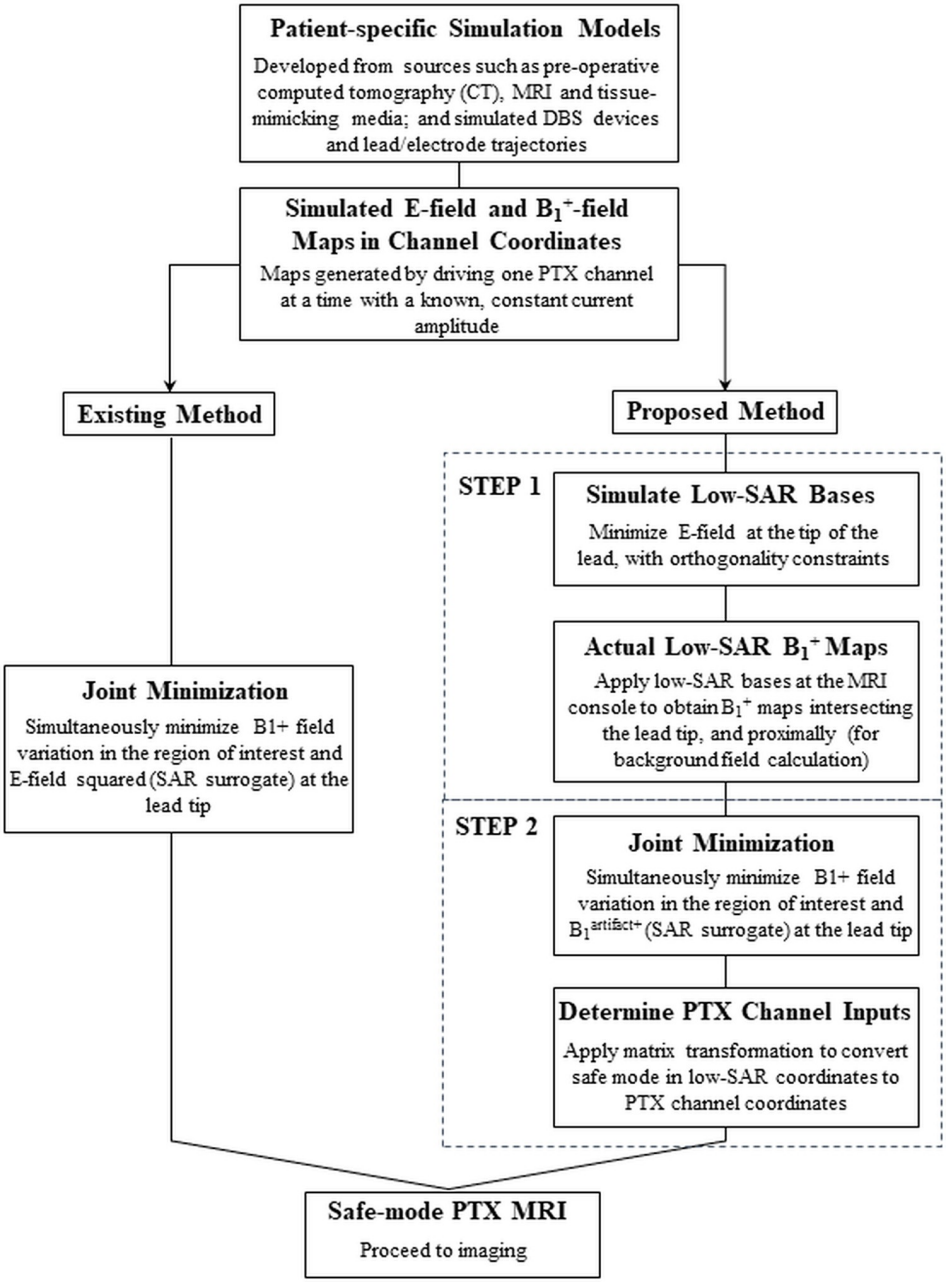
Proposed and existing workflows to obtain RF-shim PTX in safe mode. See text for details.

To first provide a brief overview of the methodology, the safe-mode workflow proposed above (briefly summarized in Fig 1) was investigated in proof of concept with a simple simulation model; and experimentally, using a phantom closely matched with the simulation model, together with fibre-optic temperature probe measurements of localized heating, and a custom-built four-channel PTX coil and research platform [22] connected to a 3 T MRI system (MAGNETOM Prisma, Siemens, Germany). For demonstration and validation purposes, the proposed workflow was compared with the existing workflow previously performed in similar studies [9–11, 22], also shown in Fig 1. The existing optimization method is solely based on simulation, thus $B_{1}^{artifact+}$ is substituted with |*E*_*tot*_|^2^ (proportional to SAR) in Eq (3) as follows:

$$\underset{{A\prime }_{m},{\varphi \prime }_{m}}{\min}\left(\frac{\sigma_{ROI}(\left|B_{1,tot}^{+}\right|)}{\mu_{ROI}(\left|B_{1,tot}^{+}\right|)}+\lambda ∙\mu_{ROM}\left({\left|E_{tot}\right|}^{2}\right)\right).$$ (7)

(Note that previous optimization studies have used either |*E*_*tot*_|^2^ [11, 22, 41] or |*E*_*tot*_| [9, 10], without major impact of choosing one or the other as the two quantities are monotonically related.) The B_1_^+^—and E—fields were simulated according to this previous method based on the basis set ***C*** (i.e., PTX channel coordinates) and used in Eq (7) to generate safe mode for application on the four-channel PTX MRI system.

In the proposed new workflow, the simulated E- maps for each channel (achieved by the basis set ***C***) were input to the first optimization function (Eqs (2A)–(2C)) to generate ***L*** low-SAR bases. The ***L*** bases were applied at the MRI console and the actual low-SAR B_1_^+^ maps were calculated for two slices (providing ${\hat{B}}_{1,tot}^{+}$ at the tip of the implant, and for estimating ${\hat{B}}_{1,coil}^{+}$ from the neighboring slice inferior to the tip). Low-SAR B_1_^+^ maps were then used in the second optimization function (Eqs (5) and (6)) on an offline computer system to obtain safe mode (*U*_*S*_) in the *U* basis, followed by matrix transformation to obtain the actual coil inputs. The results from the existing and proposed workflows were also compared with the output from a birdcage coil setup (transmit-receive (Tx/Rx) circularly polarized (CP) head coil (Siemens, Germany)), with 16 rungs. Specific details about the simulations and experiments are provided below.

### (A) Simulation model

**Fig 2:**
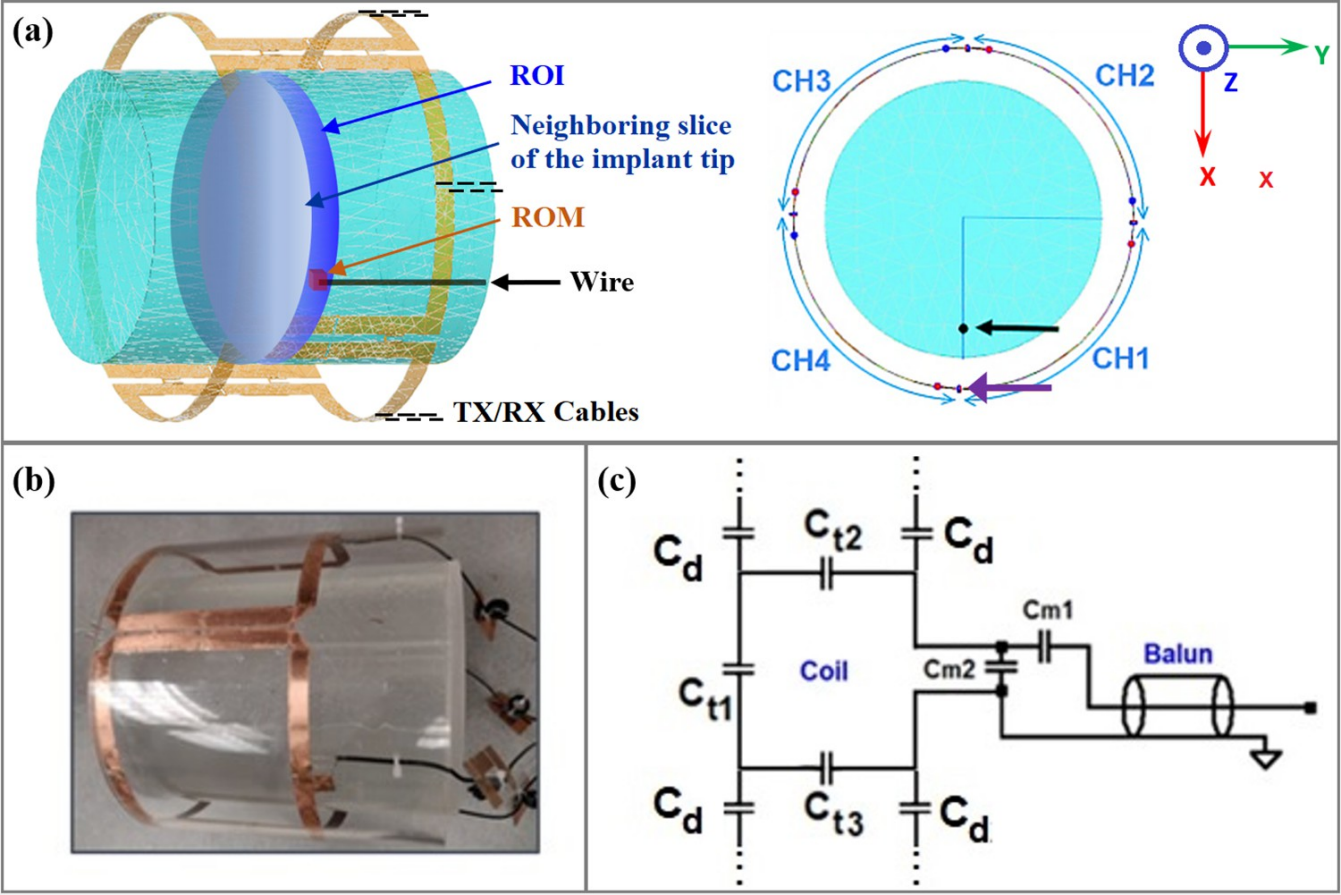
Simulation model and experimental setup. (A) The simulation model (left) for the 4-channel PTX setup of interest, including its axial cross-section in the x-y plane (right). A uniform cylindrical phantom is placed at the isocenter of the 4 coil elements (CH1 to CH4), with an implanted wire (black arrow) oriented parallel to the long axis of the cylinder (x = 6.5 cm), proximal to CH1 and CH4 with the exposed wire tip located at z = 0. Decoupling capacitors (e.g. purple arrow) are placed between neighboring coil elements to minimize crosstalk. Cables for RF transmission/reception (TX/RX) are indicated schematically by black dashed lines at the edge of each coil element. The region of minimization (ROM) is chosen as the 1 g region of tissue surrounding the implant tip (shown in red) in a circular planar region of interest (ROI, shown in dark blue). The neighboring slice of the implant tip is shown in a white-grey color gradient. (B) The experimental phantom matched with the simulation model, placed in a custom-built four channel PTX coil (the implanted wire is not visible in the shown picture) and (C) the associated electrical circuit of a single coil element, showing multiple capacitors C for tuning (C_t_), matching (C_m_) and decoupling (C_d_).

A uniform cylindrical phantom implanted with an insulated straight copper wire surrounded by a 4-channel RF transceiver coil (shown in Fig 2A) was simulated using FEKO (Altair Engineering Inc., USA). The cylinder had a radius of 9 cm; length of 24 cm; relative permittivity of 80; and conductivity of 0.47 S/m. The wire (radius of 0.5 mm; length 12 cm) was positioned 2.5 cm from the edge of the phantom with the exposed tip of 5 mm located in the axial plane at z = 0 cm. Coil elements were placed *π*/2 radians apart and had an arc length for each coil of 19.6 cm. All coils were tuned to 123.25 MHz, the resonance frequency of the hydrogen nucleus for the 3 T MRI system used in subsequent simulations and experiments, and matched to a 50 Ω source to achieve a reflection coefficient of approximately -14 dB. In addition to these diagonal elements of the scattering matrix (*S*-Matrix), the off-diagonal elements representing the various forward and reverse transmission coefficients were also calculated. The simulations were undertaken to evaluate the B_1_^+^- and E- fields produced for the ***C*** bases and ***L*** bases, using appropriately scaled voltage inputs according to the B_1_^+^ mapping experiments conducted on the MRI system (see below).

### (B) Experimental setup

The experimental setup (Fig 2B) was matched with the simulation model for the phantom composition and geometry; the copper wire design, placement within the phantom; and the placement of the phantom with respect to the PTX coil. The phantom, constructed from a poly-acrylic cylindrical pipe with closed ends, was filled with tissue-mimicking gel prepared according to ASTM International standards [35]. A fiber optic temperature probe (OTG-MPK5, Opsens, Canada) with ±0.3°C total accuracy (including both signal conditioner and sensor errors) was attached to the tip of the implant for temperature measurement. The four PTX coil elements (Fig 2C) were decoupled via capacitive decoupling, with each coil also including a balun circuit for effective power transmission from the output of the associated RF power amplifier. Using a network analyzer (E5061B, Keysight Technologies, Santa Rosa, CA, USA), all elements were tuned to 123.25 MHz, similar to the simulation model, and matched to a 50 Ω source on the lab bench to have reflection coefficients *S*_*nn*_< -14 dB while loaded with the cylindrical phantom. The various forward and reverse transmission coefficients were also recorded. A set of three MRI sessions were undertaken. In the first session, the normal automatic pre-scan procedures (i.e. to perform gradient shimming of the static magnetic field, tune to the Larmor frequency, and adjust RF transmitter power and receiver amplifier gain) were performed once with the phantom in a fixed location within the PTX coil, with all channels receiving the same small-signal input. These prescan settings were used for the subsequent comparison of B_1_^+^-maps achieved using the existing and proposed workflows, as well as to compare localized heating during B_1_^+^-mapping and also in PTX safe mode (see below). Prior to these comparison experiments, an initial calibration procedure was performed at the MRI system to measure the actual transmit magnitude and phase parameters of the PTX setup connected to the MRI system. During the calibration, incremental phases, and magnitudes to each transmit channel were applied to quantify the channel-to-channel variability. The calibrated parameters were compared to results achieved by initial testing at the benchtop. The second and third MRI sessions were undertaken with the phantom placed within the CP coil, with and without the implanted wire to provide baseline comparators of MRI signal uniformity and localized heating, respectively, with respect to the existing and proposed workflows for PTX safe mode. These sessions required new, separate auto-prescan procedures, with best efforts made to place the phantom at the same position within the CP coil in both cases.

### (C) E- and B_1_^+^ -field mapping

The EM field simulations were calculated on a 2-dimensional (2D) grid using FEKO in a specified ROM and ROI within the model, providing maps of E-field and B_1_^+^-field. A 2D grid was selected to reduce simulation time, as appropriate given the use of a straight wire and a symmetric cylindrical phantom. (This was sufficient for the present proof-of-concept work, ultimately leading to evaluation of 2D MRI data in an axial slice at the wire tip–the expected location of worst-case heating. However, a 3-dimensional (3D) grid will be necessary in the future for more complex and realistic implant trajectories.) The ROM was determined for the simulation model as the cubic 1 g region with the centroid of the cube placed at the tip of the wire. The ROM for the experimental phantom was chosen as the artifact region in close proximity to the wire tip, of similar size to that reported in the literature (i.e. approximately 1.5 mm extending from each side of the implant) [36]. A simplex optimization algorithm in Matlab (The Mathworks Inc., MA) was used to determine the complex weighting factors applied to the PTX channel inputs in the two operation modes: existing and proposed.

In the proof-of-concept MRI experiments, B_1_^+^-maps were estimated via the double angle method (DAM) [37] using a gradient-echo (GRE) sequence (repetition time TR = 2000 ms, echo time TE = 2.45 ms, slice thickness = 3 mm, acquisition matrix = 320 × 320, FOV = 220 mm, in-plane resolution = 0.68 mm x 0.68 mm) to acquire successive images (each of 5 minutes and 15 s duration) with the nominal flip angles θ_1_ and θ_2_. The normal practice using the DAM is to collect the images with θ_2_ = 2θ_1_ [37, 38], but in the present case θ_2_ = 3θ_1_ was purposefully chosen because it provides a higher signal intensity ratio (S_2_/2S_1_) than is achievable with θ_2_ = 2θ_1_, when θ_1_ < 36° (as can be calculated from the Bloch equations). This is useful under practical conditions in which the actual flip angle is substantially less than the nominal value in many regions, providing a more accurate estimate of B_1_^+^. For the existing workflow (basis vectors according to the ***C*** matrix), the same slice-selective RF pulse waveform (standard on the MRI system) was applied successively as the input to one channel while the inputs to the other channels were set to zero). For the proposed workflow (low-SAR basis vectors according to the ***L*** matrix), the amplitudes and phases for the elements of the vectors (***m1***, ***m2***, ***m3***, ***m4***) were applied to the RF pulse waveform input to each channel appropriately, additionally including corrections for the intrinsic magnitude and phase differences of each channel as determined by the initial calibration experiments (see B above). For both workflows, the DAM-GRE imaging was conducted with the flip angle of the GRE sequence set successively to θ_1_ = 20° and θ_2_ = 60°.

### (D) RF power deposition during MRI

To compare differences in localized heating produced by the ***C*** and ***L*** basis vectors in a manner that would lead to detectable temperature increases in MRI experiments, imaging was undertaken successively using two RF power-intensive turbo spin echo (TSE) sequences with the flip angles of the excitation pulses set to θ_1_ = 20° and θ_2_ = 60°, respectively; while the temperature change at the lead tip was monitored with the fibre-optic temperature probe; and with sufficient waiting time between successive image acquisitions for the phantom to cool down (acquisition matrix = 256 × 256, in-plane resolution = 0.8 mm × 0.8 mm, TR/ TE = 500 ms / 2.4 ms, slice thickness = 3 mm, and acquisition time = 5.2 mins). With the flip angle of the initial pulse remaining at 60°, a single TSE sequence was then used to conduct safe-mode MRI and compare use of the existing and proposed workflows, again while conducting the temperature measurements. For additional comparison, the analogous protocol was performed for MRI sessions two and three with the phantom placed within the CP head coil. Best efforts were made to position the phantom (with and without the wire) consistently within the coil during these two respective sessions.

## Results

Table 1 lists the *S*-Matrix scattering parameter measurements of the simulated PTX coil, and of the experimental coil at the benchtop. Reasonable agreement is shown between the two matrices, with the reflection coefficients *S*_*nn*_ along the diagonal elements remaining ≤ 14 dB in both cases. Somewhat greater differences between simulation and experiment were evident in the various forward and reverse transmission coefficients at the off-diagonal matrix elements, indicative of differences in coupling across the coil elements.

**Table 1:** Scattering parameters (*S*-Matrix) of the simulation model and the experimental bench setup using the 4-channel PTX coil configuration.

**Channel Number**		**Simulation Model [dB]**				**Benchtop Test [dB]**			
	**1**	**-13**	**-27**	**-13**	**-50**	**-17**	**-25**	**-16**	**-18**
	**2**	**-27**	**-13**	**-28**	**-13**	**-25**	**-14**	**-30**	**-16**
	**3**	**-13**	**-28**	**-13**	**-27**	**-16**	**-30**	**-15**	**-30**
	**4**	**-50**	**-13**	**-27**	**-13**	**-18**	**-16**	**-30**	**-18**
		**1**	**2**	**3**	**4**	**1**	**2**	**3**	**4**
	**Channel Number**								

Table 2 lists tuning, matching, and decoupling capacitors for each element of the coil; the *S*_*nn*_ values (repeated from Table 1 for ease of comparison) and phases for each of the four channel elements with the coil loaded by the phantom on the benchtop; as well as the *S*_*nn*_ values and phases for the PTX setup calibrated with the coil connected to the MRI system. Of note, the *S*_*nn*_ values were consistently < -10 dB for the MRI calibration, indicating effective power transmission. Although the MRI calibration *S*_*nn*_ and phase values were found to be stable throughout a single experimental session, it was found in preliminary experiments that the values might vary from one session to another due to factors affecting the coil loading, such as the presence of the wire implanted in the phantom, slight changes in the coil and phantom positioning, temperature fluctuations, mismatching between simulation and experimental transmission amplitudes and phases, as well as the amplitude and phase errors of the RF amplifiers (±0.5 dB and ±10°, respectively). As mentioned in the Methods section, the subsequent PTX results were thus obtained from a single experimental session, in which the MRI calibration values in Table 2 were found to be appropriate and stable.

**Table 2:** Calibration capacitors (see Fig 2C), phases for the benchtop test, and *S*_*nn*_ scattering parameters with analogous phase MRI calibration values for each element of the 4-channel PTX coil configuration.

**Tuning, Matching and Decoupling Capacitors (pF)**		**CH1**	**CH2**	**CH3**	**CH4**
	***C***_***t*1**_	28.0	21.2	12.0	21.4
	***C***_***t*2**_	50.6	10.0	13.9	10.0
	***C***_***t*3**_	29.6	10.0	12.0	10.0
	***C***_***m*1**_	10.0	28.6	25.0	26.5
	***C***_***m*2**_	21.02	22.8	25.0	20.2
	***C***_***d***_	8.2			
**Benchtop Test**	***S***_***nn***_ **(dB)**	-17	-14	-15	-18
	**Phase (°**)	74	114	150	121
**MRI Calibration**	***S***_***nn***_ **(dB)**	-10	-12	-13	-18
	**Phase (°**)	220	0	180	80

**Fig 3:**
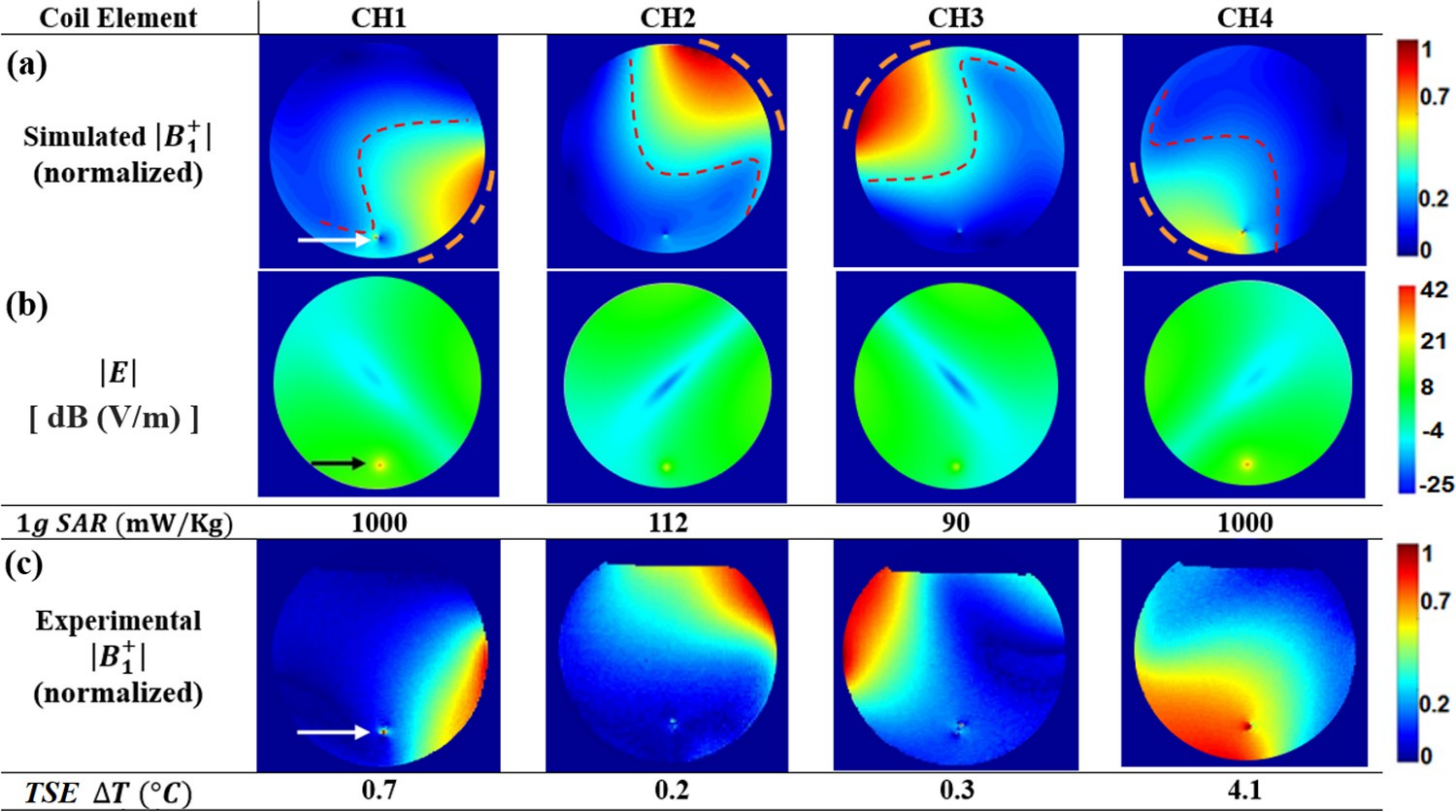
|B1+| and |E| maps using the standard basis. **(**A**)** Simulated normalized |B_1_^+^| maps for the model of Fig 2, using the standard basis (“channel coordinates”), with orange dashed lines indicating the approximate position of the active element, for coil channels CH1, CH2, CH3 and CH4. Normalization was performed with respect to the maximum |B_1_^+^| observed across all four channels. The “twisted” red dashed line highlights the field asymmetry due to the elliptical field polarization effect (see text for details). **(**B**)** Corresponding simulated |E|-fields on a logarithmic scale. The 1-g local SAR at the tip of the wire is shown below each |E| map. **(**C**)** Corresponding normalized |B_1_^+^| maps estimated by MRI experiment from two gradient echo images using the double angle method (DAM) with flip angles set to θ_1_ = 20° and θ_2_ = 60°. The measured temperature change at the wire tip during TSE MRI, ΔT, is shown below each experimental normalized |B_1_^+^| map. The wire tip is indicated via white and black arrows in the |B_1_^+^| and |E| maps, respectively.

Fig 3 shows summary results from simulations, experimental MRI data and thermometry following the existing approach for PTX MRI, which involves B_1_^+^ mapping in channel coordinates. Simulated maps of the normalized B_1_^+^- and the associated E-field magnitudes are reported in Fig 3A and 3B, respectively, in the axial plane of the medium, located at the wire tip. For ease of comparison between simulation and experiment (as well as with the analogous results using the low-SAR bases, reported below) the spatial dependency of each magnitude map of |B_1_^+^| is shown normalized with respect to the maximum signal value observed across each of the PTX channels. The E-field magnitudes are reported in dB to compress the dynamic range of the results for better visualization. As anticipated, Fig 3A shows normalized |B_1_^+^| spatial profiles that were localized to each of the four channel elements CH1-CH4, with maximal values close to (and values that decrease with distance from) a given element. Interestingly, Fig 3A also shows that the transmit field pattern was “twisted” (see red dashed line), such that the pattern was not symmetric with respect to the orthonormal line from the centre of an element (placement of the elements around the phantom is indicated by orange dashed lines). In free space, a surface loop coil produces a linearly polarized B_1_-field which is decomposed into equal contributions of right (B_1_^+^) and left (B_1_^-^) circular polarization, producing a symmetric field pattern [39]. In a conductive sample, the applied B_1_-field induces a current which generates an out-of-phase magnetic field which adds location-dependent contributions to the overall B_1_-field, yielding an elliptical polarization–consistent with the twisting patterns shown. This phenomenon has been explained well in a previous study [39]. In addition, a field perturbation due to RF coupling was evident at the wire tip location (white arrow) for each channel, with larger amplitude observed for the channels that were more proximal to the wire (CH1 and CH4). In Fig 3B, the main |E| dependence observed was that each channel element showed a zone of reduced field extending radially in the circular cross-section of the medium, along the orthonormal line from the centre of the element, and with minimal magnitude observed near the centre of the medium. The RF coupling effect was also observed as an E-field perturbation in each case with the analogous dependency as observed for the normalized |B_1_^+^| data in Fig 3A (i.e., stronger coupling and elevated |E| for CH1 and CH4 compared to CH2 and CH3). To quantify these coupling effects, the 1-g local SAR value at the wire tip is reported below each simulated |E| map in Fig 3B, with values of 1000 mW/Kg obtained for CH1 and CH4, and values of 112 and 90 mW/Kg for CH2 and CH3, respectively. Fig 3C shows the experimental equivalent of Fig 3A, depicting normalized |B_1_^+^| maps for each coil channel as obtained using the DAM to image the phantom and PTX coil setup that was matched to that of the EM simulations. When using the TSE sequence to transmit on each channel in analogous fashion, the temperature increases at the wire tip, ΔT, were found to be 0.7, 0.2, 0.3 and 4.1°C, respectively.

The localized nature of the normalized |B_1_^+^| maps obtained experimentally was very similar to that of the simulated results for each coil element, with some discrepancies that were likely due to imperfect matching between simulation and experiment, as described previously. In particular, the normalized |B_1_^+^| maps obtained experimentally showed fields that were more localized to the surface of the phantom, in comparison to the simulation results, for CH1-CH3, whereas the opposite effect was shown for CH4. As shown in Table 2, the MRI calibration *S*_*nn*_ value was -18 dB for CH4, whereas it was -10 dB, -12 dB, -13 dB for the other CH1, CH2 and CH3, respectively. Although best efforts were made to minimize the channel-to-channel variability in the initial calibration, achieving the most optimal controlled experimental set up was challenging due to the presence of the wire and the “twisted” |B_1_^+^| pattern of CH4 close to the wire, which was not the case for any of the other coil elements. Given these differences, it is not surprising that there were also differences in the simulated |B_1_^+^| perturbation and associated experimental |B_1_^+^| artifact for each coil channel–qualitatively, however, the simulated and experimental results were still similar.

**Fig 4:**
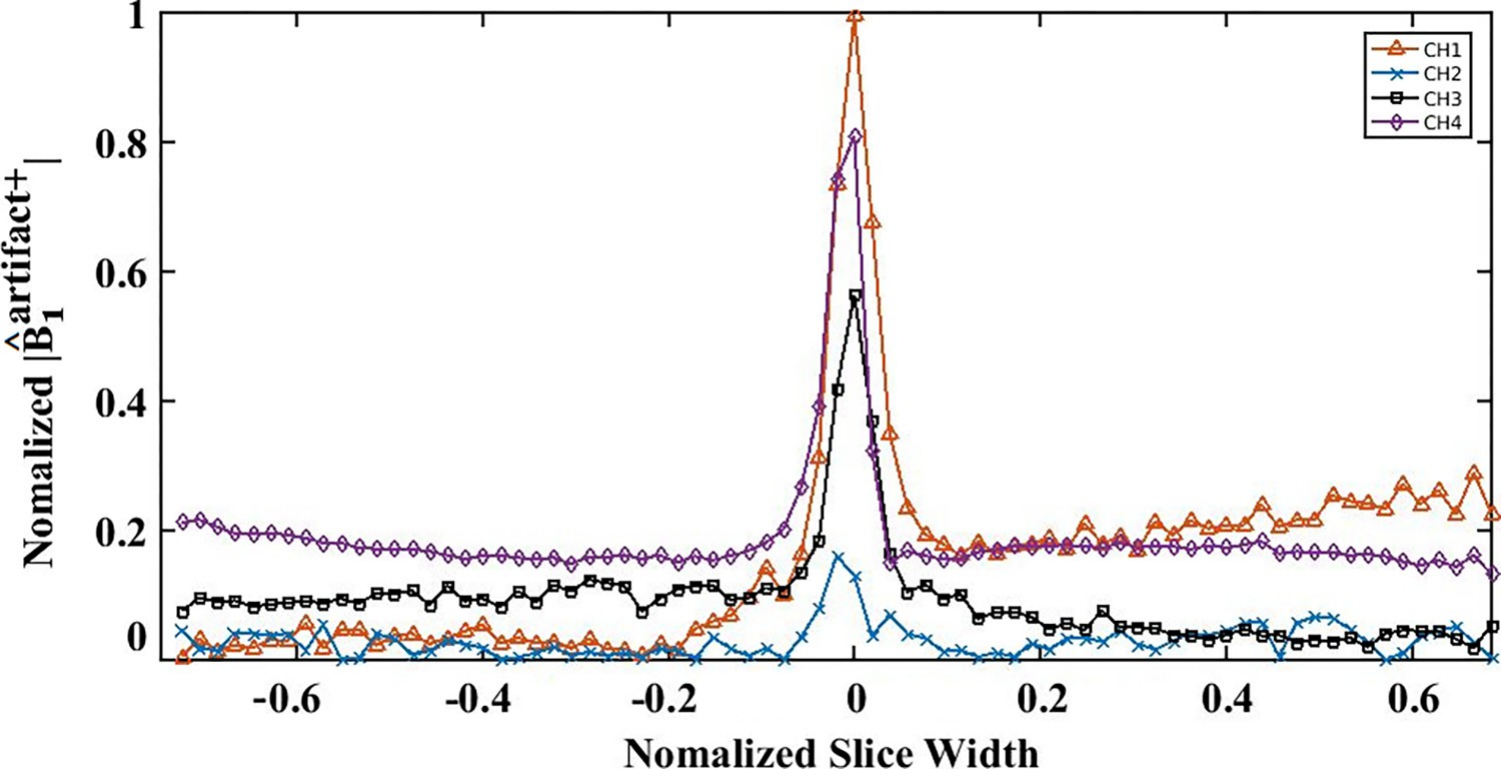
Line plots of |${\hat{B}}_{1}^{artifact+}$|. The plots are taken horizontally through a plane intersecting the wire location, as represented by the |B_1_^+^| maps for CH1-CH4 as shown in Fig 3C. The artifact (normalized to the maximum value observed across channels) surrounding the wire tip is much larger for CH1 and CH4 (elements closer to the wire) than for CH2 and CH3 (elements further from the wire).

Fig 4 shows line plots of |${\hat{B}}_{1}^{artifact+}$| values (calculated based on Eq (4) and normalized to the maximum value obtained across channels) for CH1 to CH4. Each value was extracted from Fig 3C, with the lines taken horizontally in the axial images to intersect with the wire location. From these plots it is evident that during the experiment, the RF coupling artifacts for CH1 and CH4 were much larger than those of CH2 and CH3. This result was expected, as the latter elements were more remote from the wire. Furthermore, this ranking also agrees with the temperature changes measured experimentally for the individual coil elements during TSE MRI, as shown below the images in Fig 3C. The temperature rise was substantially higher for CH1 and CH4 (0.7° and 4.1°, respectively) compared to the negligible values for CH2 and CH3 (0.2° and 0.3°, respectively, within the ±0.3° experimental error of the temperature probe).

However, some discrepancies were also observed between the localized heating in the simulations, and the temperature changes obtained experimentally. Whereas the 1-g local SAR results given in Fig 3B were large and equal for CH1 and CH4, and the normalized |B_1_^+^| artifacts were quite similar, the temperature change during the experiments was much larger for CH4 (4.1°C) than for CH1 (0.7°C). In addition, the normalized |B_1_^+^| artifact was larger for CH3 than for CH2, whereas both channels showed negligible temperature elevation as reported above.

**Fig 5:**
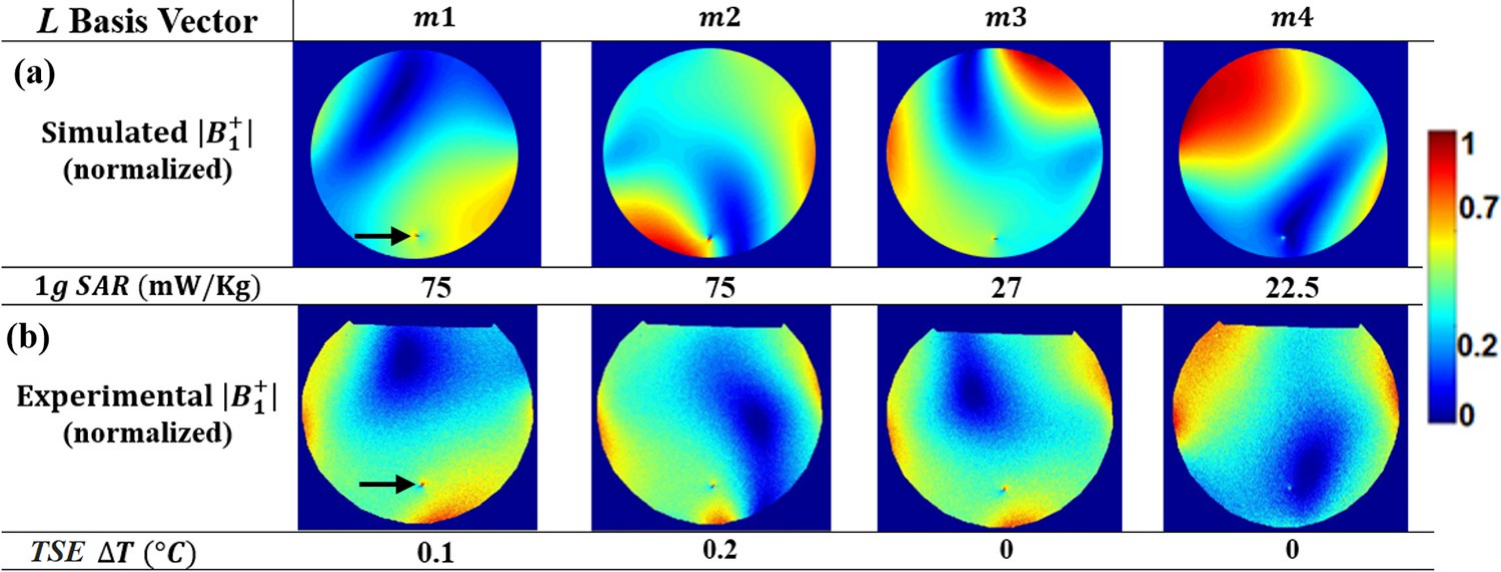
Low-SAR |B1+| maps. Simulated **(**A**)** and analogous experimental **(**B**)** normalized |B_1_^+^| maps for the low-SAR ***L*** basis vectors of Table 3, respectively. The wire tip is indicated via a black arrow. The 1-g local SAR at the tip of the wire, as obtained from E-field simulations, is shown below each simulated |B_1_^+^| map. The measured temperature change at the wire tip during TSE MRI, ΔT, is shown below each experimental normalized |B_1_^+^| map.

Using the E-maps simulated in channel coordinates, shown in Fig 3B, simulated ***L*** matrix bases were then calculated for step 1 of the proposed new workflow using Eqs (2A)–(2C). These bases are listed in the top half of Table 3 and the corresponding simulated low SAR normalized |B_1_^+^| maps are shown in Fig 5A, with the 1-g local SAR surrounding the wire tip determined as 22.5 (***m4***), 27 (***m3***) and 75 mW/Kg (***m1*** and ***m2***). To indicate whether the SAR level for the simulated E-maps achieved by the ***C*** and ***L*** bases was within the safety guidelines set by the US Food and Drug Administration (FDA), the corresponding values of the peak local 1-g SAR (SAR_1g_) and 10-g SAR (SAR_10g_) were calculated for the wire location, as well as the global SAR (SAR_G_). The ratios *SAR*_1*g*_/*SAR*_*G*_ and *SAR*_10*g*_/*SAR*_*G*_ were also calculated, with all values listed in Table 4. The FDA safety regulation requires *SAR*_1*g*_/*SAR*_*G*_ < 2.7; Table 4 shows that this condition was not met for the ***C*** bases (CH1 and CH4 achieved a ratio of 4.9, with CH2 and CH3 at 0.58 and 0.47, respectively) but was met for the ***L*** bases (1.74 for ***m1*** and ***m2***, with ***m3*** and ***m4*** at 0.64 and 0.50, respectively).

**Table 3:** The *L* matrix basis vectors (*m1*, *m2*, *m3*, *m4*) applied to 4-channel PTX for Step 1 of the proposed low-SAR workflow.

	*m*1	*m*2	*m*3	*m4*
	Amplitude, Phase (°)			
Simulated ***L*** bases	0.35, 150.9	0.40, 73.3	0.22, 34.5	0.52, 101.3
	0.52, 101.3	0.22, 34.5	0.40, 73.3	0.35, 150.9
	0.40, 73.3	0.35, 150.9	0.52, 101.3	0.22, 34.5
	0.22, 34.5	0.52, 101.3	0.35, 150.9	0.40, 73.3
Post-Calibration ***L*** bases	0.32, 10.9	0. 45, 293.3	0.2, 254.5	0.52, 321.3
	0.52, 101.3	0.31, 34.5	0.42, 73.3	0.4, 150.9
	0.38, 253.3	0.38, 330.9	0.52, 281.3	0.24, 214.5
	0.2, 114.5	0.52, 181.3	0.3, 230.9	0.38, 153.3

**Table 4:** Values for 1-g SAR (SAR_1g_), 10-g local SAR (SAR_10g_) and global SAR (SAR_G_), as well as *SAR*_1*g*_/*SAR*_*G*_ and *SAR*_10*g*_/*SAR*_*G*_ ratios calculated for the simulated E- maps, as achieved with the *C* (*CH1*, *CH 2*, *CH 3*, *CH 4*) and *L* (*m1*, *m2*, *m3*, *m4*) bases.

SAR	CH1	CH2	CH3	CH4	*m*1	*m*2	*m*3	*m*4
SAR_1g_ (W/Kg)	1	0.112	0.09	1	0.075	0.075	0.027	0.022
SAR_10g_ (W/Kg)	0.249	0.026	0.022	0.252	0.0187	0.0178	0.0062	0.0053
SAR_G_ (W/Kg)	0.203	0.19	0.19	0.203	0.043	0.043	0.042	0.042
SAR_1g_/SAR_G_	4.9	0.58	0.47	4.9	1.74	1.74	0.64	0.5
SAR_10g_/SAR_G_	1.23	0.14	0.118	1.28	0.435	0.414	0.148	0.125

To obtain analogous low-SAR maps in actual MRI experiments for comparison, the channel-dependent calibration values (Table 2) were applied appropriately to the simulation ***L*** bases to obtain post-calibration ***L*** bases for use at the MRI console (Table 3, bottom). The resulting normalized |B_1_^+^| maps for each ***L*** basis (Fig 5B) were generated using the DAM method and experimental setup as described above. Note that the relative amplitudes of the elements in each ***L*** basis were scaled appropriately prior to PTX, such that the maximum magnitude of B_1_^+^ was identical during each mapping procedure: in this case equalling 3.1 μT. The simulated and experimental |B_1_^+^| maps look similar for each basis in Fig 5A and 5B and compared to the respective maps in Fig 3A and 3C, the zones of minimal |B_1_^+^| values show much less regional extent. Moreover, the temperature increases during TSE MRI were 0.1, 0.2, 0 and 0°*C* for ***m*1** to ***m*4**, respectively–markedly less than those obtained in channel coordinates (Fig 3C).

Turning to step 2 of the proposed workflow, several optimizations were initially performed across a range of λ values to investigate how the regularization parameter affected joint minimization of the |B_1_^+^| artifact in the ROM (localized heating) and minimization of B_1_^+^ inhomogeneity in the ROI. Optimizations were conducted over the range from λ = 0 (exclusively minimizing B_1_^+^ homogeneity) to λ = 1 (balanced B_1_^+^ inhomogeneity and artifact terms in Eq (5)). The associated simulations were conducted at a nominally low spatial resolution for expediency with the resulting optimized amplitude and phase values used to assess 1-g SAR at the wire tip. Table 5 lists the optimized amplitude and phase solutions, |B_1_^+^| artifact within the ROM (expressed as the percentage increase from background), B_1_^+^ inhomogeneity within the ROI, and corresponding 1-g SAR across the range of λ values. The results indicate that although the relationships between λ and |B_1_^+^| artifact and between λ and B_1_^+^ inhomogeneity are not linear, larger values of λ broadly correspond to smaller |B_1_^+^| artifact and 1-g SAR values, at the cost of moderately increased B_1_^+^ inhomogeneity in comparison to what is achievable with λ = 0.

**Table 5:** Application of various λ values in Step 2 of the proposed low-SAR Workflow.

*λ*	CH1	CH2	CH3	CH4	|B_1_^+^| Artifact (%)	B_1_^+^ inhomogeneity (%)	SAR_1g_ (mW/Kg)
	Amplitude, Phase (°)						
0	0.75, 130	1.0, 116	0.5, 313.5	0.55, 291	56	21	5000
0.35	0.21, 90	1.0, 267	0.75, 296	0.41, 351	43	21	450
0.75	0.77, 318	0.31, 345	0.55, 0	0.41, 298	32	50	112
0.90	0.31, 345	0.58, 0	0.47, 318	1.0, 298	10	52	80
1.0	0.23, 73	0.73, 295	0.85, 231	0.38, 321	12	40	50

**Fig 6:**
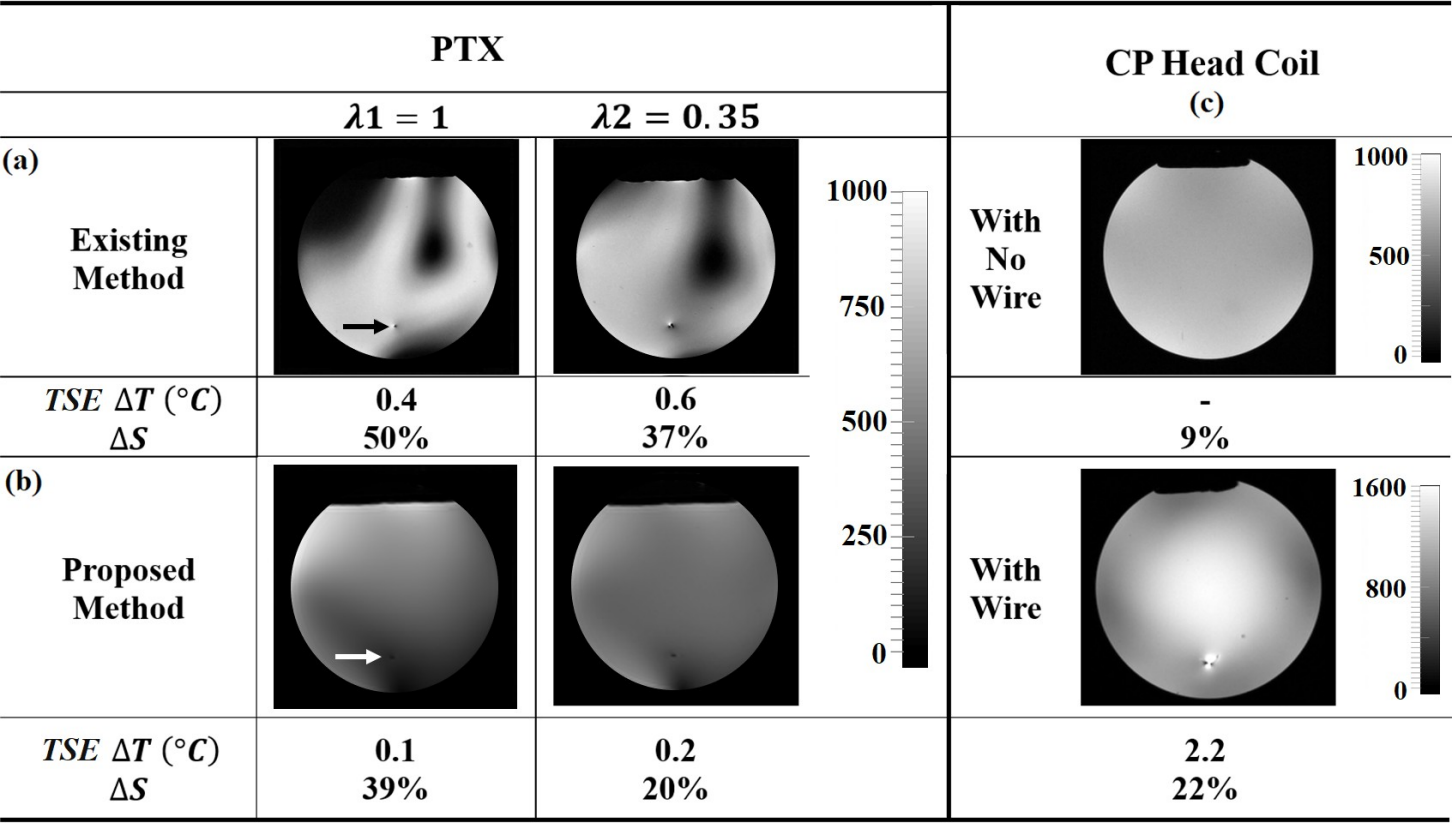
Workflow comparison. Safe-mode PTX MRI with channel settings determined using the existing **(**A**)** and proposed **(**B**)** workflows, for two different regularization parameter values (*λ*1 = 1 and *λ*2 = 0.35). Results are shown in **(**C**)** for MRI using a circularly polarized (CP) head-coil transceiver, for the same phantom setup, without and with the implanted wire. The measured temperature increase during TSE MRI, Δ*T*, and the signal intensity variation, Δ*S*, across the phantom are shown below each image. The wire tip is indicated via a black or white arrow.

The final results compare the PTX safe mode images, heating and signal nonuniformity obtained by the existing and newly proposed workflows (Fig 1), together with the data obtained using the standard CP head coil. For the existing workflow for safe mode, the simulated B_1_^+^ and E maps (represented in Fig 3A and 3B) were applied to the optimization function in Eq (7), for two different regularization values (*λ*1 = 1 and *λ*2 = 0.35) to illustrate the effect on the results of penalizing B_1_^+^ nonuniformity more or less. The resulting RF shim values (post-calibration for application at the MRI console) are listed in Table 6 and the respective safe-mode PTX images are shown in Fig 6A. Temperature increases of ΔT = 0.4 and ΔT = 0.6° C were detected from TSE MRI, and the signal intensity variations, ΔS (standard deviation/mean across the total axial plane area of the phantom intersecting at the wire location, expressed as a percentage) were calculated as 50% and 37% for *λ*1 and *λ*2, respectively. For the proposed workflow to obtain safe mode, the set of four low-SAR B_1_^+^ maps (represented in Fig 5B) were applied to optimization step 2 using the same *λ*1 and *λ*2 values (Eqs (5)–(6)). The optimized shim settings were transformed to channel coordinates and the calibration adjustments were applied, resulting in the values listed in the bottom half of Table 6. The matrix condition number for the transformation was calculated to be 4.2. The respective images acquired experimentally for *λ*1 and *λ*2 are shown in Fig 6B, with ΔT = 0.1 and ΔT = 0.2°C from TSE MRI, and ΔS = 39% and ΔS = 20%, respectively. For both *λ*1 and *λ*2 values, the proposed workflow produced less temperature increase and minimal signal intensity artifact proximal to the wire tip, and less signal intensity variation than was obtained with the existing workflow. For additional comparison, the ΔS value increased from 9% to 22% when TSE MRI was conducted with the CP head coil and the wire was added to the phantom; the artifact at the tip of the wire was also much larger in extent and of higher amplitude than observed for either PTX MRI solutions–as was the localized heating, measured as ΔT = 2.2°*C*. These results are shown in Fig 6C.

**Table 6:** Optimized post-calibration amplitude and phase shifts for 4-channel PTX in safe mode, for two different regularization parameters.

	*λ*1 = 1		*λ*2 = 0.35	
*Channel*	Magnitude	Phase (°)	Magnitude	Phase (°)
***Existing Workflow (Optimize in Channel Coordinates)***				
***1***	0.95	0	0.65	80
***2***	1	75	0.7	75
***3***	0.65	8.1	0.35	8.1
***4***	0.73	151	0.43	212
***Proposed Workflow (Optimize in low-SAR Coordinates)***				
***1***	0.23	73	0.2	89
***2***	0.73	295	1	267
***3***	0.85	231	0.75	296
***4***	0.38	321	0.41	351

## Discussion

The present work proposes and investigates a new workflow for PTX safe mode with RF shimming. The new workflow, despite having some additional complexity compared to the existing workflow, offers a number of advantages: 1) improved B_1_^+^ mapping, by producing maps with smaller zones of low signal intensity, in low-SAR coordinates (Step 1); 2) use of such experimentally acquired maps, rather than simulated B_1_^+^ maps, in the joint minimization procedure to generate safe mode (Step 2); 3) use of the actual |B_1_^+^| artifacts as a surrogate of local RF power deposition, replacing the simulated E-field term in the joint minimization to estimate safe mode (Step 2); and 4) production of safe-mode PTX images with enhanced signal uniformity over the FOV while maintaining (or perhaps improving) suppression of localized heating effects in a model system involving an implanted wire. These advantages, as well as the limitations of the present work are discussed below.

Regarding the use of |B_1_^+^| artifact rather than |*E*| or |*E*|^2^ in the joint optimization, a simple monotonic relationship was not observed between |B_1_^+^| artifact and either of the other two quantities, but it was confirmed that the two coil elements closest to the implanted wire showed the largest |B_1_^+^| artifacts and localized SAR levels. Furthermore, it is logical that minimal RF coupling leads to minimal concentrated E fields, localized heating and |B_1_^+^| artifact at the wire tip. Minimizing (and ideally eliminating) the |B_1_^+^| artifact should therefore lead to effective suppression of localized heating–as was observed in these proof-of-concept experiments.

Comparing the PTX safe mode results from the two workflows (Fig 6) in more detail, the most obvious effect was that the improved signal uniformity produced by the proposed workflow came from the elimination of large signal voids (indicative of undesirable phase cancellation effects). The voids observed with the existing workflow were likely a reflection of imperfect matching of the simulations with the actual experimental conditions, as will be discussed further below. The voids were less apparent when the regularization parameter λ was adjusted to place more emphasis on minimizing B_1_^+^ inhomogeneity when estimating safe mode. However, this improvement came at the expense of slightly increased localized heating. The dependency of safe mode results on λ values was not tested exhaustively, but based on the present results it is reasonable to speculate that there is unlikely to be a scenario where the existing workflow performs better than the proposed workflow.

In addition, the proposed workflow led to lower safe-mode MRI signal amplitudes in Fig 6 than those of the existing workflow. This effect–a nominal reduction of 20%—likely arose because the existing workflow produced not only signal voids (phase cancellations) but also enhancements (phase coherences) due to imperfect RF shimming. Furthermore, the B_1_^+^ field can be intensified by RF coupling effects, as demonstrated in Fig 6 by standard MRI of the phantom with and without the implanted wire, using the CP coil. The proposed workflow thus likely produced an improved RF shim result by effectively suppressing both phase effects and coupling effects.

The validity of the existing workflow has been previously investigated through multiple studies that yielded positive results with heterogenous phantoms, unilateral and bilateral curved implanted wires [10, 40] and realistic patient DBS lead-trajectories obtained from intra-operative CT data [11]. The new proposed workflow enhances the robustness of the existing workflow, and thus promising results are expected by extension for other PTX coils, complex phantom geometries, and implant designs and trajectories that are representative of those used clinically. However, given the proof-of-concept nature of the present work, future studies will be needed to confirm this speculation conclusively–showing that the low-SAR bases can be determined robustly under such conditions.

Several methodological details of the present work also warrant discussion. Good agreement was observed between simulated and experimental results for the normalized B_1_^+^ maps in channel coordinates and in low-SAR coordinates, respectively, although localized discrepancies were evident as well. The discrepancies were likely due to imperfections in the matching of the simulated PTX coil components and phantom geometry, with their actual counterparts used in the MRI experiments–as characterized by the *S*-matrix values listed in Tables 1 and 2. In addition, the cylindrical phantom used in the MRI experiments had a sizeable air bubble that changed the boundary conditions for the RF electromagnetic fields compared to the perfect cylinder model used in the simulations. In channel coordinates, the latter issue had most impact on the experimental results for the CH2 and CH3 basis vectors when these channels were excited in isolation, as the CH2 and CH3 coil elements were located closest to the air bubble. In the experiments conducted in low-SAR coordinates, the fields for all basis vectors were affected, given that each basis generated wide-ranging B_1_^+^- and E-fields throughout the phantom. Notably, the proposed new workflow utilized the actual B_1_^+^ maps obtained experimentally whereas the existing workflow relied only on the simulations, which was likely an important factor contributing to why the former case gave improved results in PTX safe mode. Ultimately, it will be important in the future to develop increasingly robust workflows to enable PTX safe mode MRI in patients, that hopefully have minimal reliance on EM simulations and permit rapid setup at the MRI system console. The present work constitutes an initial step in this direction.

In the present work, the choice was made to conduct all comparisons between the existing workflow and the proposed new workflow for PTX safe mode with the MRI system tuned to the same set of auto-prescan values–of which the fixed transmitter power setting is especially notable. This led to differences in |B_1_^+^| maps between the two experimental conditions. For clarity, Table 7 lists the mean and maximum values of the simulated and experimental |B_1_^+^| maps in the ROI from Fig 3A and 3C for the ***C*** bases (CH1, CH2, CH3 and CH4), as well as from Fig 5A and 5B for the ***L*** bases (***m1***, ***m2***, ***m3*** and ***m4***). Additionally, the corresponding 1-g local SAR and the measured temperature change (ΔT) at the wire tip during each TSE DAM (with θ_1_ = 20° and θ_2_ = 60°) are shown below each ***C*** and ***L*** bases, respectively. For the simulation results, the mean (maximum) |B_1_^+^| values in the ROI for the simulated ***C*** bases were 0.13 (0.58), 0.12 (0.47), 0.13 (0.46), and 0.12 (0.71) μT for CH1, CH2, CH3 and CH4, respectively. In comparison, for the ***L*** bases, the values were 0.06 (0.16), 0.051 (0.16), 0.053 (0.15), and 0.06 (0.16) μT for ***m1***, ***m2***, ***m3*** and ***m4***, respectively. When using the DAM, in the experimental setup, the mean |B_1_^+^| values for the flip angle set to θ_1_ = 20° were 0.20 (CH1), 0.27 (CH2), 0.24 (CH3) and 0.47 μT (CH4) for the ***C*** bases, compared to 0.10 (***m2*** and ***m4***) and 0.12 μT (***m1*** and ***m3***) for the ***L*** bases. For θ_2_ = 60°, the mean |B_1_^+^| values were 0.95 (CH1), 1.24 (CH2), 1.12 (CH3) and 1.5 μT (CH4) for the ***C*** bases, and 0.54 (***m1***), 0.53 (***m2***), 0.57(***m3***) and 0.47 μT (***m4***) for the ***L*** bases. Note that for the simulations and experiments, the mean |B_1_^+^| values for the ***L*** bases were approximately half the value for the ***C*** bases, with the notable exception that the mean |B_1_^+^| values for CH4 were skewed larger due to particularly strong RF coupling with the wire. An additional observation is that the ratio of mean |B_1_^+^| values (for θ_2_/θ_1_) for a given basis vector (***C*** or ***L***) were in the range of 4–5, markedly exceeding the expected value of 3.0. This is likely due to the variable impact of zones of very low signal intensity in the |B_1_^+^| maps, which particularly affected the averaging operation over the ROI when θ_1_ = 20°. Maximum |B_1_^+^| values for θ_1_ were highest for the ***C*** bases for CH4 at 2.9 μT, with values for the ***L*** bases peaking at 0.77 μT in ***m1*** and ***m2***. For θ_1_, the maximum |B_1_^+^| value for the ***C*** bases reached 6.18 μT in CH4, compared to a maximum of 3.1 μT in the ***L*** bases for ***m1*** and ***m2***. Given these data and especially the large maximum |B_1_^+^| values observed for CH4, it is evident why the decision was made to evaluate the |B_1_^+^| maps with separate normalizations for each basis in Figs 3–5, for ease of interpretability–rather than normalizing to the maximum |B_1_^+^| value obtained across both bases. Lastly, it is reiterated in Table 7 that the temperature changes (ΔT) at the wire tip were more pronounced in the ***C*** bases, with ΔT values of 0.3°C to 2.5°C, compared to minimal changes in the ***L*** bases.

**Table 7:** Mean and maximum |B1+| values calculated over the Region of Interest (ROI) for the simulated and experimental |B1+| maps, and associated temperature elevations ΔT as achieved with the *C* (*CH1*, *CH 2*, *CH 3*, *CH 4*) and *L* (*m1*, *m2*, *m3*, *m4*) bases shown in the Figs 3 and 5, respectively. θ_1_ and θ_2_ refer to the flip angles used during the Double Angle Method used for B_1_^+^ mapping.

		CH1	CH2	CH3	CH4	*m*1	*m*2	*m*3	*m*4
**Simulation**	Mean |B_1_^+^| (μT)	0.13	0.12	0.13	0.12	0.06	0.051	0.053	0.06
	Max |B_1_^+^| (μT)	0.58	0.47	0.46	0.71	0.16	0.16	0.15	0.16
	SAR_1g_ (mW/Kg)	1000	112	90	1000	75	75	27	22.5
**Experiment**	Mean |B_1_^+^| for *θ*_*1*_ (μT)	0.20	0.27	0.24	0.47	0.12	0.10	0.12	0.10
	Mean |B_1_^+^| for *θ*_*2*_ (μT)	0.95	1.24	1.12	1.5	0.54	0.53	0.57	0.47
	Max |B_1_^+^| for *θ*_*1*_ (μT)	1.42	1.64	1.97	2.9	0.77	0.77	0.70	0.50
	Max |B_1_^+^| for *θ*_*2*_ (μT)	3.6	4.5	4.1	6.18	3.1	3.1	2.9	2.2
	ΔT for *θ*_*1*_ *(*^*o*^*C)*	0.3	0.1	0.1	1.6	0	0.1	0	0
	ΔT for *θ*_*2*_ *(*^*o*^*C)*	0.4	0.1	0.2	2.5	0.1	0.1	0	0

Other controlled experimental conditions could also have been undertaken: for example, by obtaining B_1_^+^ maps and PTX safe mode MRI results for both workflows using a matched, spatially-averaged flip angle within a pre-defined zone within the phantom; or possibly by using matched total transmit power. The matched flip angle approach is potentially complicated by uncertainty in determining *a priori* what zone to specify for matching the flip angle, given that signal non-uniformity can significantly impact the results when comparing safe mode or B_1_^+^ mapping results (e.g. as shown in Figs 3, 5 and 6). Furthermore, in the context of PTX RF shimming, matching the flip angle does not guarantee that the same power is transmitted to the sample or patient, nor does it guarantee the same E-field conditions at the tip of a conductive implant. By extension, matching in terms of the total power transmitted by the PTX amplifiers is complicated according to the same argument: the low-SAR bases, and the RF shimming results for PTX safe mode using both workflows, depend on the vector summation of the magnitudes and phases of the electromagnetic fields generated by the inputs to all the transmit channels. Thus, the total transmit power is not a reliable proxy of global or local SAR in the context of this PTX scenario.

Irrespective of whether there is an “optimal” controlled experimental protocol for the present work, the methodology that was followed enabled important conclusions to be drawn: The mean and maximum |B_1_^+^| values across the four channels of the simulated ***C*** bases were relatively consistent, however, the 1-g local SAR value at the wire tip varied significantly, with CH1 and CH4 (elements closer to the wire) exhibiting much higher SAR compared to CH2 and CH3 (elements further from the wire), as expected. Comparing the simulated ***C*** and ***L*** bases: the mean (maximum) |B_1_^+^| values in CH1 to CH4 are 2 times (3 to 4.4 times) larger than those in the ***L*** bases while the ratio of the SAR_1g_ values for CH1 to CH4 are 13.3, 1.5, 3.3, and 44 times greater than those for ***m1***, ***m2***, ***m3*** and ***m4***, respectively. These SAR_1g_ ratios should also match the reduction in 1-g volume-averaged |E^2^| when adopting the ***L*** basis versus the ***C*** basis. It is possible that some of this power reduction could be due to mismatch between the B_1_^+^ magnitudes (and by extension, E magnitudes) when adopting the ***L*** basis versus the ***C*** basis in the simulations and experiments. However, considering the worst-case scenarios, the ***C*** basis for CH4 was investigated at |B_1_^+^| = 6.18 μT whereas the analogous value for ***m1*** and ***m2*** was 3.1 μT. Assuming a linear relationship between maximum B_1_^+^ and E magnitudes, this equates to a factor of 4 in power reduction. Thus, given that 4 << 13.3 and 44, these simple calculations suggest that the majority of the reduction in 1-g SAR (and by extension, localized heating as confirmed in the TSE experiments) produced by the ***L*** bases is due to the optimization procedure undertaken in step 1 of the proposed workflow (local SAR minimization by RF shimming intrinsic to each basis vector), and not the mismatch in transmit power.

An additional methodological issue of interest related to the constraints placed on inputs to the PTX channels when performing step 1 of the proposed new workflow to obtain the low-SAR bases. A lower bound of 0.2 was set for the relative amplitude of any channel to avoid the possible scenario that low power transmission from one or more channels in relation to the others would increase the potential for zones of low B_1_^+^ amplitude and subsequent noise contamination in B_1_^+^ maps. This was found to be prudent, as the ***L*** matrix used in the experiments (Table 3) had single elements for low-SAR bases ***m1*** and ***m3*** that reached this lower bound. A relative amplitude of 1.0 was chosen as the upper bound to constrain the matrix condition number and thus propagation of any experimental errors in determining (***m1***, ***m2***, ***m3***, ***m4***) into PTX safe mode MRI. The condition number obtained for ***L***^***-1***^ was 4.2, indicating a modest level of error amplification in the outcome, although promising results were obtained irrespectively in terms of markedly improved signal uniformity and suppression of localized heating in PTX safe mode for both regularization parameter values, compared to the results obtained with the existing workflow. These results apply to the existing experimental scenario, and more exhaustive exploration of the optimization parameter space, in terms of constraints on ***m*** vectors, the matrix condition number, and regularization parameter values would be required when extending the low-SAR basis approach to more realistic conditions and towards applications in DBS patients.

Another interesting speculation about low-SAR basis vectors is also evident in retrospect. As a starting point, Full-PTX pulse designs have previously been developed in a joint numerical optimization framework that enables SAR considerations to be included as terms in the cost function [13, 14]. However, if the Full-PTX pulses are designed in the low-SAR basis, the concerns over localized heating can be taken into consideration at the time of B_1_^+^ mapping. Thus, the need for joint minimization is reduced at the time of RF pulse design, and also the need for the user to explore the regularization parameter space and establish the best trade-off between terms in the cost function. These intriguing possibilities are under active investigation in the laboratory.

Some limitations of the proposed workflow are also evident. First, the channel inputs, particularly phase settings, had to be determined quite carefully by manual adjustment to obtain the best suppression of localized heating effects–irrespective of whether the existing or proposed workflow was followed. This places emphasis on careful phase calibration of PTX hardware prior to commencing safe-mode workflows. Interestingly, a previous simulation study suggested that an 8-channel setup may be more robust to errors in channel settings, presumably as the impact of error in a given channel has less contribution to the overall outcome [41]. A rapid, automatic phase calibration procedure should be developed in the future.

Second, although the present work showed proof of concept, it did not establish a workflow for clinical implementation of PTX safe mode MRI. A clinical implementation would require several additional modifications. In particular, a more efficient and practical method of B_1_^+^ mapping would need to be adopted. The DAM was adopted here for its simplicity to perform experimental validation, using a slow GRE approach for convenience, followed by TSE MRI and localized temperature measurements to demonstrate explicitly that the low-SAR bases suppressed localized heating at the wire tip better than was achieved by implementing safe mode PTX with the existing workflow in channel coordinates. Given the potential risks of permanent tissue damage posed by localized heating, it is reasonable to develop approaches such as the low-SAR bases that strike an acceptable balance between increased complexity in workflow to generate safe mode, while taking an ALARA approach to localized heating. Furthermore, a clinical implementation would likely adopt an accelerated B_1_^+^ mapping method at lower transmission power than used here, for even greater safety. In this context, the additional benefit of the low-SAR bases—i.e., improved B_1_^+^ mapping due to smaller zones of low signal intensity, and thus improved calculation of PTX safe mode—thus would be very useful. Possible alternatives for B_1_^+^ mapping in a clinical implementation include actual flip angle imaging [42], Look-Locker methods [43, 44], and “calibration-less” PTX which reconstructs transmit B_1_^+^ maps from under-sampled k-space transmit and receive data in seconds/slice [45].

Third, further investigations are likely required concerning the joint minimization of B_1_^+^ nonuniformity and localized SAR. In the present work, the joint minimization was revised to involve the |B_1_^+^| (RF coupling) artifact as a surrogate of local SAR, rather than |*E*|^2^ or |*E*|. Although this approach improved the quality of the results when compared to those of the existing workflow, two additional aspects will require investigation in future work. First, the use |*E*|^2^ versus |*E*|, as well as the revision to |B_1_^+^|, will affect the relative weighting between the two terms jointly minimized, as adjusted by the regularization parameter λ. Thus, given the influence of chosen λ values on safe mode results as shown in Table 5, some of the differences in PTX safe mode MRI results for the existing and proposed workflow as shown in Fig 6 may be due to the different impact of the fixed λ choices in the two minimization strategies. A detailed investigation of the impact of λ values on PTX safe mode results for the existing workflow (joint minimizations involving |*E*|^2^ and |*E*| terms) and for the proposed workflow should be conducted in the future. Second, the joint minimization used in the present work focuses on the ROM, but in the scenario involving DBS patients, does not address whether local SAR hotspots will be generated elsewhere in the head by the optimal RF shim settings. The proof-of-concept EM simulation data shown here for 1-g, 10-g and global SAR indicated that no additional hotspots were evident for a simple wire and cylinder model, but other simulations suggest that this will not necessarily be the case when PTX safe mode is applied in clinical DBS situations [10]. The generation of the low-SAR bases is also subject to the same concern, especially as this step in the proposed new workflow still relies on estimating E-fields using EM simulations. Local SAR control over the entire head is highly desirable but is known to be very computationally demanding when explored solely by EM simulations, due to the need for an exhaustive search over all head sub-volumes during the optimization procedure [46]. One way to mitigate this problem in the future would be to make use of the Virtual Observation Points (VOP) method [46], a search compression tool that can be used in combination with PTX pulse optimization to minimize SAR matrices into smaller sets, reducing computing time and maximizing accuracy in identifying local SAR maxima. For example, VOP have previously been used in optimized RF “spoke” pulse design to control SAR in regions outside of a DBS device [13].

Fourth, this study employed a straight insulated wire with an exposed tip for proof of concept. However, modern DBS devices are typically designed with multiple electrodes featuring intricate geometric configurations to enhance control and precision in targeting the E-field distribution within the brain [47]. Furthermore, the associated DBS leads often incorporate helical wires to improve flexibility. Research indicates that straight DBS leads generate significantly more heat than helical leads [48, 49], making the use of straight wires in this study a valid conservative case for proof of concept. Moreover, such studies [49] demonstrate that the electrical and thermal RF responses of single-electrode and multi-electrode leads differ significantly. For instance, the temperature distribution near the contacts of multi-electrode leads is asymmetric, leading to an uneven distribution of hot spots around the electrodes. One potential solution to determine safe-mode solutions reliably in real patients with such devices may be to define the major dimension of the ROM based on the distance between the first and last contacts (including some expansion for safety considerations). In the case of the Medtronic 3387 model, for example, the dimension would be >10.5 mm (given that each contact length is 1.5 mm, with a 1.5 mm spacing between the four adjacent contacts). However, further investigation is required to refine the modeling of multi-electrode leads, ensuring that both the lead and electrode configurations are accurately considered. As part of such work, it will be interesting to determine whether the same (suitably chosen) PTX safe mode parameters can be used for different DBS devices with the same lead trajectory and electrode target–and, the extent to which safe mode parameters can be relaxed in the case of newer DBS devices that have been engineered to exhibit less heating than legacy devices during MRI.

In conclusion, this study demonstrates a new, robust workflow to determine optimal complex inputs to individual PTX coil elements towards safe MRI of patients with DBS implants, with improved B_1_^+^ uniformity and without deleterious localized heating. The workflow produces promising results in a simple, proof-of-concept matched simulation model and experimental system; and provides an important step toward clinical implementation by introducing improved B_1_^+^ mapping using orthogonal bases of PTX settings that keep heating as low as reasonably achievable.

## Supporting information

S1 Table Temperature measurement at the wire tip during TSE MRI using the standard bases (“Channel coordinate”), low-SAR bases and safe-mode PTX MRI with channel settings determined in the existing and proposed workflows. (XLS)

S1 File MATLAB codes for determining complex weighting factors applied to PTX channel inputs in two operation modes. (ZIP)

S2 File The channel-dependent relative amplitudes (A_n_), and the corresponding calculated in logarithmic scale (A_n_ = 20 × log (S_n_(t)_out_ / S_n_(t)_in_) where S_n_(t)_in_ = 1). (XLS)

S3 File Simulated E-Field maps using the standard basis (“channel coordinates”) and the low-SAR *L* basis vectors of Table 3. (ZIP)

S4 FileSimulated B_1_^+^ maps using the standard and the low-SAR bases and the experimental ${\hat{B}}_{1}^{artifact+}$ maps for CH1-CH4.(ZIP)

S5 File B_1_^+^-mapping using the DAM calculated from the Bloch equations based on *θ*_2_ = 2*θ*_1_ and θ_2_ = 3θ_1_. (DOCX)

S1 Fig Signal ratio *R*/2 = (*S*2/2*S*1) computed using Equation S3 in S1 File for successive application of a gradient echo pulse sequence with flip angles *θ*_1_ and *θ*_2_, where *θ*_2_ = 2*θ*_1_ (red) and *θ*_2_ = 3*θ*_1_ (blue). The latter condition produces larger R/2 values for *θ*_1_ < 36°.
(TIF)
