# Supplementary material for: Optimized radiofrequency shimming using low-heating B1+-mapping in the presence of deep brain stimulation implants: Proof of concept
Source: PLoS One. 2024 Dec 18;19(12):e0316002. doi: 10.1371/journal.pone.0316002 (PMC11654958; doi:10.1371/journal.pone.0316002)
Supplement: S5 File — (DOCX) [file pone.0316002.s006.docx]

The signal ratio S_2_/S_1_, denoted as R, is given by:

$$R=\frac{S_{2}}{S_{1}}=\frac{\sin\theta_{2}}{\sin\theta_{1}}. (S1)$$

The signal ratio from two variations of flip angles is then given by:

$$R=\left\{ \begin{aligned} \frac{2 sin \theta_{1}\cos\theta_{1}}{\sin\theta_{1}}, if \theta_{2}=2\theta_{1} \\ \frac{3\sin\theta_{1}-4\sin^{3} \theta_{1}}{\sin\theta_{1}}, if \theta_{2}=3\theta_{1}. \end{aligned} \right. (S2)$$

Equation S2 can be simplified to:

$$\frac{R}{2}=\left\{ \begin{aligned} \cos\theta_{1}, if \theta_{2}=2\theta_{1} \\ \frac{3}{2}-2\sin^{2} \theta_{1}, if\theta_{2}=3\theta_{1}. \end{aligned} \right. (S3)$$

Using $\theta_{1}=\gamma B_{1}t$, the transmit B_1_ - ﬁeld is thus given by:

$$B_{1}^{+}=\left\{ \begin{aligned} \frac{1}{\gamma t}\cos^{-1} \left( \frac{R}{2} \right), if \theta_{2}=2\theta_{1} \\ \frac{1}{\gamma t}\sin^{-1} \left( \frac{\sqrt{3-R}}{2} \right), if \theta_{2}=3\theta_{1}. \end{aligned} (S4) \right.$$

Figure S1 illustrates the signal ratio curves for Equation S3 in the DAM. It is apparent that for $\theta_{1}$ < $36^{\circ}$, $R/2$ has greater amplitude in the latter case than in the former. For $\theta_{1}=20^{\circ}$:

$$\frac{R}{2}=\left\{ \begin{aligned} 0.86, if \theta_{2}=40^{\circ} \\ 1.26, if \theta_{2}=60^{\circ}, \end{aligned} (S5) \right.$$
