# Supplementary figures and images for: Optimized radiofrequency shimming using low-heating B1+-mapping in the presence of deep brain stimulation implants: Proof of concept
Source: PLoS One. 2024 Dec 18;19(12):e0316002. doi: 10.1371/journal.pone.0316002 (PMC11654958; doi:10.1371/journal.pone.0316002)

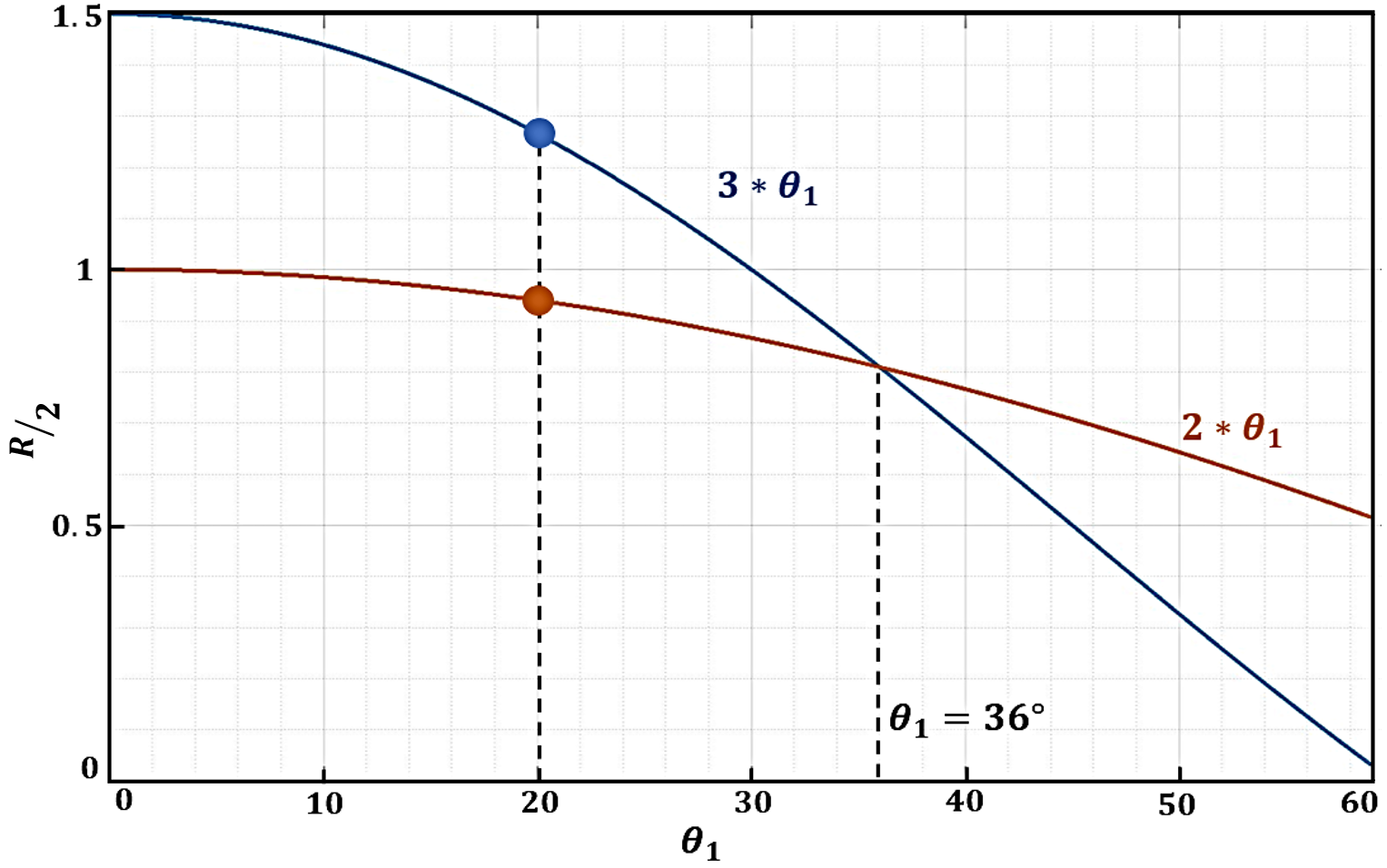

Supplement: S1 Fig — The latter condition produces larger R/2 values for θ1 < 36°. (TIF) [file pone.0316002.s007.tif]
